# Expression Profiling of Strawberry Allergen Fra a during Fruit Ripening Controlled by Exogenous Auxin

**DOI:** 10.3390/ijms18061186

**Published:** 2017-06-02

**Authors:** Misaki Ishibashi, Hiroki Yoshikawa, Yuichi Uno

**Affiliations:** Department of Plant Resource Science, Graduate School of Agricultural Science, Kobe University, Rokko, Kobe, Hyogo 657-8501, Japan; 151a302a@stu.kobe-u.ac.jp (M.I.); 177a321a@stu.kobe-u.ac.jp (H.Y.)

**Keywords:** allergen, auxin, auxin inhibitor, *Fragaria* × *ananassa*, oral allergic syndrome, pathogenesis-related, phytohormone, post-harvest, pre-harvest, ripening regulation

## Abstract

Strawberry fruit contain the allergenic Fra a proteins, members of the pathogenesis-related 10 protein family that causes oral allergic syndrome symptoms. Fra a proteins are involved in the flavonoid biosynthesis pathway, which might be important for color development in fruits. Auxin is an important plant hormone in strawberry fruit that controls fruit fleshiness and ripening. In this study, we treated strawberry fruits with exogenous auxin or auxin inhibitors at pre- and post-harvest stages, and analyzed Fra a transcriptional and translational expression levels during fruit development by real-time PCR and immunoblotting. Pre-harvest treatment with 1-naphthaleneacetic acid (NAA) alone did not affect Fra a expression, but applied in conjunction with achene removal NAA promoted fruit pigmentation and Fra a protein accumulation. The response was developmental stage-specific: Fra a 1 was highly expressed in immature fruit, whereas Fra a 2 was expressed in young to ripe fruit. In post-harvest treatments, auxin did not contribute to Fra a induction. Auxin inhibitors delayed fruit ripening; as a result, they seemed to influence *Fra a 1* expression. Thus, Fra a expression was not directly regulated by auxin, but might be associated with the ripening process and/or external factors in a paralog-specific manner.

## 1. Introduction

Strawberry (*Fragaria* × *ananassa*) is a popular fruit consumed worldwide. Strawberry fruit are rich in secondary metabolites, which contribute to enhanced nutritional value for humans, such as antioxidant activities [1]. The fruit also contains an allergen that triggers symptoms in individuals that suffer from oral allergy syndrome (OAS). Symptoms of OAS include itching, tingling, and swelling in the mouth or throat. The Fra a proteins are a major allergen group identified in strawberry [2]. The Fra a proteins consist of several isoforms encoded by many *Fra a* genes [3,4,5]. Sixteen sequences corresponding to *Fra a* genes have been identified in diploid strawberry *Fragaria vesca* based on genomic sequence information [6,7]. In the case of octoploid cultivated strawberry (*F.* × *ananassa*), many more *Fra a* genes may exist. To breed hypoallergenic strawberry, it is difficult to eliminate such a large number of genes by cross-breeding. However, a gene recombination technique can cope with such a circumstance. Muñoz et al. [4] reported that RNA interference technology was successful in transiently down-regulating *Fra a* genes. However, the sale and/or outdoor cultivation of genetically modified organisms is not permitted in some countries. Indeed, in Japan, genetically-modified strawberry implanted with interferon genes is grown within an enclosed secure facility termed a plant factory. When growing fruit crops in a plant factory, pollination may be problematic. The pollination of strawberry is mostly reliant on honeybees or bumblebees [8], but there are difficulties in controlling pollination in an artificial environment or achieving a sufficient supply of pollinators. An alternative solution is parthenocarpy, which has been utilized in some fruit and vegetable crops, such as tomato and eggplant [9,10]. Parthenocarpic fruit set in these plants is accomplished by spraying open flowers with auxin. Auxin is an important phytohormone in strawberry that controls fruit set and development, as well as fruit fleshiness and ripening. As an endogenous auxin, indole-3-acetic acid (IAA) is biosynthesized in the achenes and subsequently affects receptacle development [11]. When utilizing auxin for parthenocarpy, it is important to pay attention to the inducibility of undesirable genes. During the fruit ripening process, several gene expression patterns are altered by auxin [12].

The Fra a allergens belong to the pathogenesis-related (PR) 10 protein family [13,14]. The PR proteins are associated with defense mechanisms, including responses to biotic/abiotic stresses [15,16]. Phytohormones often regulate PR-10 expression in the signal transduction pathway in response to biotic/abiotic stresses [17]. The gene *Bet v 1*, which is an ortholog of *Fra a* in birch, is induced by exogenous auxin in birch roots [18]. Expression of *PR-10* genes in *Zea mays* is regulated by plant hormone treatments; in particular, *ZmPR10.1* is downregulated by 1-naphthaleneacetic acid (NAA) [19]. These findings raise the possibility that *Fra a* expression is influenced by auxin. As a PR-10 defense mechanism, Fra a 1 protein is induced by UV-C treatment in cultivated strawberry [20]. In roots of wild strawberry, the expression of allergen-like genes is raised by pestilence bacteria (*Phytophthora cactorum*) [21]. In addition, several flavonoids were shown as the ligands of the Fra a proteins [22]. Knowledge of the expression patterns of Fra a genes and proteins in response to auxin treatments of strawberry is of importance for down-regulation of the allergenic proteins. Moreover, if application of exogenous auxin or an auxin inhibitor regulates both *Fra a* expression and fruit ripening, it may represent a useful cultivation technique for the production of hypoallergenic strawberry. In this study, we analyzed the differential expression of Fra a paralogs during fruit development, and in response to pre- and post-harvest treatment with auxin and auxin inhibitors. We also discuss practical approaches to reduce the accumulation of Fra a allergens in strawberry fruit.

## 2. Results

### 2.1. Fruit Morphology and Response of Fra a to Pre-Harvest Auxin Treatments

**Figure 1:**
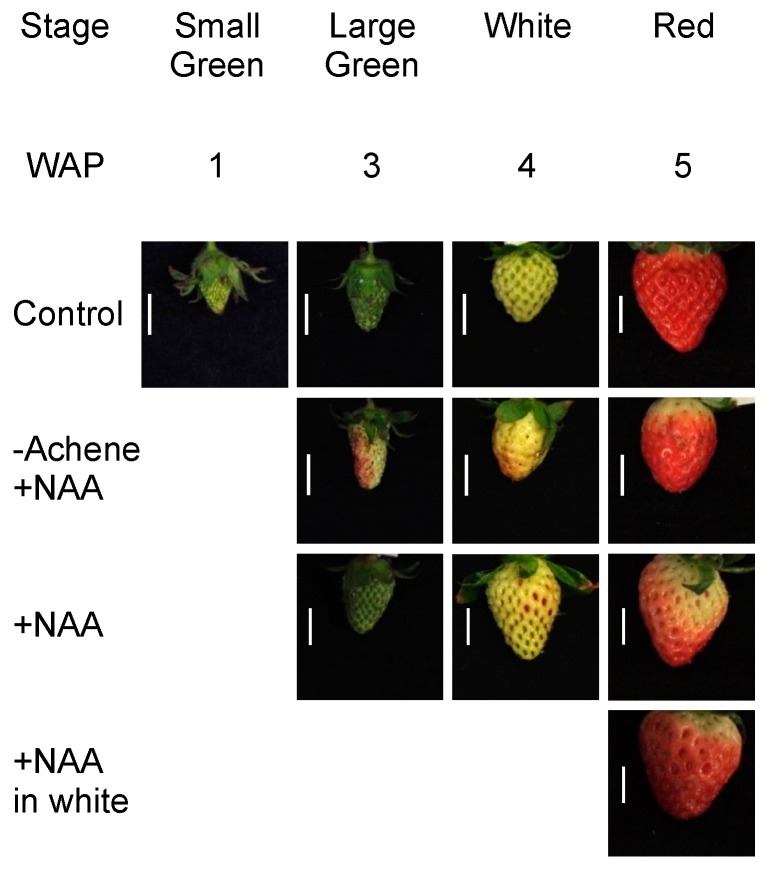
Development of strawberry (*Fragaria* × *ananassa* cv. “Akihime”) fruit in response to pre-harvest treatment with auxin. Strawberry plants were grown in containers in a glasshouse. Approximate number of weeks after artificial pollination (WAP) is shown above the photographs. The developmental stages were defined as small green, large green, white, and red. The surface of the fruit was pasted with lanolin emulsion, which was durable for long-term application. The lanoline emulsion contained solvent alone (control) or 3000 µM 1-naphthaleneacetic acid (+NAA). All treatments were performed at the small green stage except for one treatment applied at the white stage (+NAA in white). Achenes were removed to exclude the influence of endogenous auxin on receptacle development (−Achene+NAA). Scale bar represents 1.0 cm.

The surface of strawberry fruits was pasted with lanolin emulsion durable for long-term usage as a pre-harvest treatment (Figure 1). The lanoline emulsion was applied with solvent alone (control) or containing 3000 µM NAA (+NAA). No significant differences in the maturity (L*, a*, and b* scores) and growth (LD, TD, and FW) of fruit between the control and +NAA treatment were observed at the red stage of fruit development (Figure 1 and Table 1). When NAA treatment was applied at the white stage (+NAA in white), no significant difference was observed from NAA treatment at the small green stage (+NAA). The removal of achenes to exclude the influence of endogenous auxin (−Achene+NAA) promoted pigmentation at the large green stage, but significantly decreased the transverse diameter of fruit at the white stage. Pigmentation was notable in the apical portion of the fruit not covered by the calyx. In the absence of NAA treatment, achene removal completely inhibited receptacle development (Figure S1). These results indicated that NAA effectively stimulated fruit pigmentation and growth, which were otherwise inhibited by achene removal.

**Figure 2:**
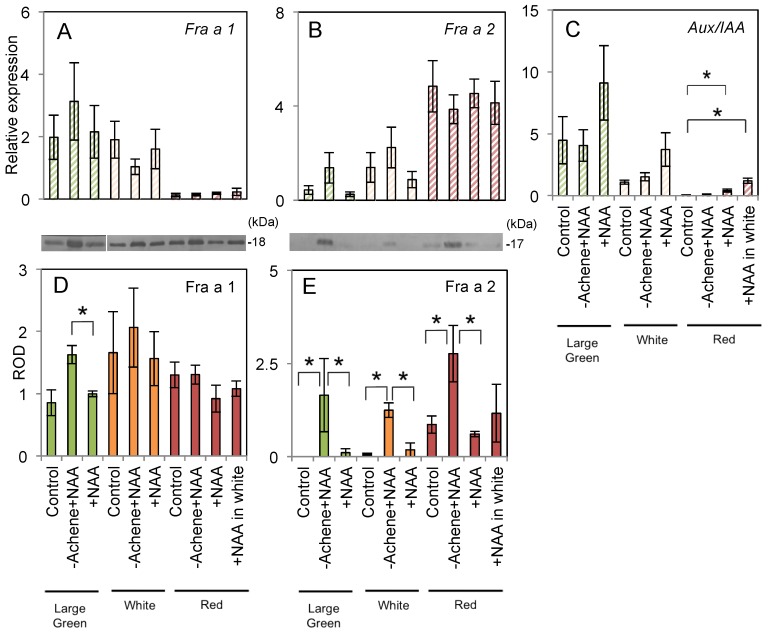
Expression of Fra a genes and proteins in response to pre-harvest treatment with auxin at various developmental stages in the fruit of strawberry (*Fragaria × ananassa* cv. “Akihime”). Culture and pre-harvest treatments were performed as described in Figure 1. RNA and protein were extracted from fruits harvested at the large green (green columns), white (orange columns), and red (red columns) stages. The relative transcript levels of *Fra a 1* (**A**), *Fra a 2* (**B**), and *FaAux/IAA* (**C**) were detected by real-time PCR and normalized against that of the elongation factor 1α gene (*EF1α*). Fra a proteins were detected by immunoblotting with guinea pig antisera, including a polyclonal antibody raised against 6× His-tagged recombinant Fra a 1e (**D**), or a rabbit antibody raised against a Fra a 2 peptide (**E**). Optical densities were quantified using Image J (1.50i, National Institutes of Health, USA) from original blots shown above the graph. Relative optical densities (ROD) were calculated from normalized values against the control. Asterisks indicate significant differences between the same color columns (Wilcoxon test, *n* = 4, *p* < 0.05).

For real-time PCR analysis of *Fra a* gene expression, *Fra a 1* and *Fra a 2* were targeted as major paralogs. In the control, expression of *Fra a 1* decreased (Figure 2A), while *Fra a 2* expression increased (Figure 2B), as fruit development proceeded. This finding is consistent with a previous study by Muñoz et al. [4]. No significant difference was observed among the control and each auxin treatment for both *Fra a* paralogs. The transcript level of *FaAux/IAA*, as an indicator gene for auxin response, differed significantly between the control and the “+NAA” and “+NAA in white (rightmost bar)” treatments (Figure 2C). Therefore, pre-harvest treatment with NAA did not directly induce expression of *Fra a 1* and *Fra a 2*. We analyzed Fra a translational products at the three fruit ripening stages by immunoblotting. A significant difference in Fra a 1 accumulation was observed between the ”+NAA” and ”−Achene+NAA” treatments at the large green stage (Figure 2D). Accumulation of Fra a 2 in “−Achene+NAA” fruits was significantly induced at each developmental stage (Figure 2E). Especially at the green and white stages, Fra a 2 detection was notable in the “−Achene+NAA” treatment. This response did not correspond with the results for gene transcripts. In regression analysis, the Fra a mRNA and protein expression levels were not correlated (Fra a 1: *R*^2^ = 0.00; Fra a 2: *R*^2^ = 0.19).

### 2.2. Fruit Morphology and Response of Fra a to Pre-Harvest Auxin-Inhibitor Treatments

**Figure 3:**
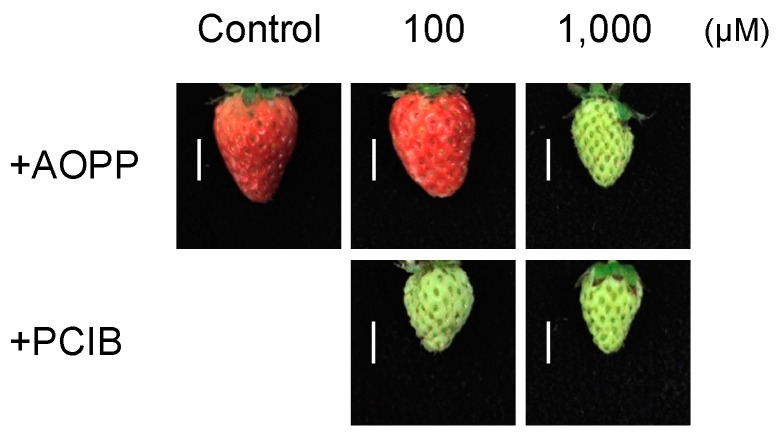
Development of strawberry (*Fragaria* × *ananassa* cv. “Akihime”) fruits in response to pre-harvest treatment with auxin inhibitors. Culture and pre-harvest treatment were performed as described in Figure 1. The fruit surface was pasted with lanoline emulsion containing solvent alone (control), l-2-aminooxy-3-phenylpropionic acid (+AOPP), or *p*-chlorophenoxyisobutyric acid (+PCIB) at concentrations of 100 (100) or 1000 µM (1000) approximately one week after pollination at the small green stage. The fruit were harvested at approximately 5 WAP. Scale bar represents 1.0 cm.

To investigate the inhibition of metabolism and transduction pathways associated with auxin, we applied two chemical compounds as auxin inhibitors: L-2-aminooxy-3-phenylpropionic acid (AOPP) and *p*-chlorophenoxyisobutyric acid (PCIB). Fruit at the small green stage were treated with a lanoline emulsion containing AOPP or PCIB at concentrations of 0 (control), 100 (100), or 1000 µM (1000) and harvested at approximately five weeks after pollination (WAP). Fruit maturation was affected in a dose-dependent manner, as shown in Figure 3, which was confirmed by colorimetric quantification (Table 2). The a* scores of AOPP- and PCIB-treated fruit were significantly lower than that of the control. This finding is not consistent with a previous study by Symons et al. [23] and may reflect the difference in the fruit developmental stages at which the treatment was applied. Fruit growth did not differ significantly among the treatments (Figure 3 and Table 2).

**Figure 4:**
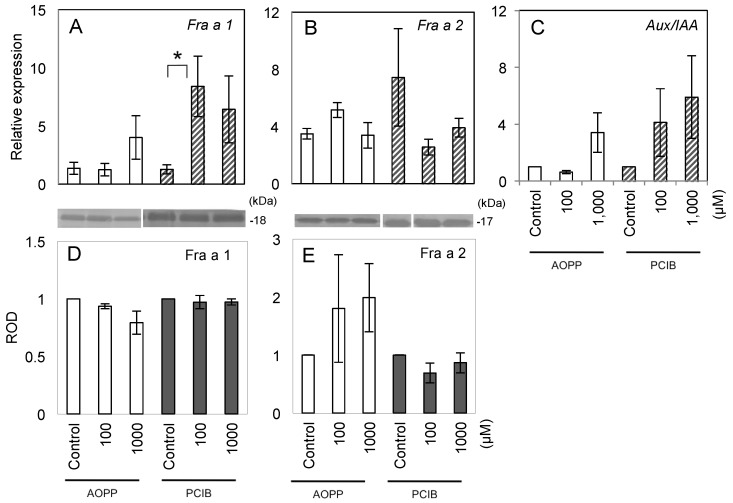
Expression of Fra a genes and proteins in response to pre-harvest treatment with auxin inhibitors at various developmental stages in fruit of strawberry (*Fragaria × ananassa* cv. “Akihime”). Culture and pre-harvest treatments were performed as described in Figure 3. RNA and protein were extracted from harvested fruit treated with the auxin inhibitors l-2-aminooxy-3-phenylpropionic acid (AOPP; open columns) and *p*-chlorophenoxyisobutyric acid (PCIB; shaded columns). The relative transcript levels of *Fra a 1* (**A**), *Fra a 2* (**B**), and *FaAux/IAA* (**C**) were detected by real-time PCR, as described in Figure 2. Both Fra a 1 (**D**) and Fra a 2 (**E**) proteins were detected by immunoblotting, as described in Figure 2. An asterisk indicates significant difference between the same color columns (Wilcoxon test, *n* = 4, *p* < 0.05).

In addition, we analyzed Fra a and *FaAux/IAA* expression in fruit treated with the auxin inhibitors. *Fra a 1* expression was significantly induced in fruit treated with 100 µM PCIB (Figure 4A). No significant differences were observed in both relative expression of *Fra a 2* and Fra a protein accumulation among each treatment with AOPP and PCIB (Figure 4B,D,E). The expression levels of *FaAux/IAA* did not differ significantly (Figure 4C). These results indicated that Fra a expression was not regulated by auxin at both transcriptional and translational levels, as suggested by the responses to pre-harvest auxin treatments. In regression analysis, the Fra a 1 mRNA and protein expression levels were not correlated (*R*^2^ = 0.01) and Fra a 2 was weakly correlated (*R*^2^ = 0.27).

### 2.3. Expression of Fra a Protein in Response to Post-Harvest Auxin Treatments

**Figure 5:**
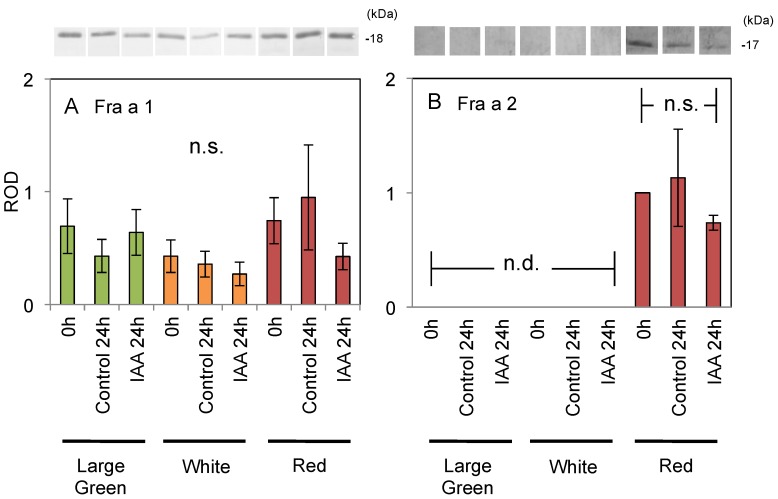
Expression of Fra a proteins in response to post-harvest treatment with auxin at various developmental stages in the fruit of strawberry (*Fragaria × ananassa* cv. “Akihime”). Fruit were harvested at the large green (green columns), white (orange columns), and red (red columns) stages. Harvested fruit were dipped in 0.3% ethanol solvent alone (control 24 h) or 3000 µM indole-3-acetic acid solution (IAA 24 h). Both Fra a 1 (**A**) and Fra a 2 (**B**) proteins were detected by immunoblotting as described in Figure 2. “n.s.” and “n.d.” indicate “not significant” and “not detected”, respectively. Significant differences were not detected between treatments at each developmental stage (Wilcoxon test, *n* = 4, *p* < 0.05).

To analyze the short-term response of Fra a protein accumulation to exogenous auxin, fruit were dipped directly in IAA solution as a post-harvest treatment. Red fruit were comparably softened after 24 h’ treatment without deformation or color changes. No significant differences were observed among the three treatments at each developmental stage. Fra a 1 accumulated consistently in fruit at each stage (Figure 5A), whereas Fra a 2 accumulation was only detected in red fruit (Figure 5B). These data confirmed that Fra a accumulation in the fruit was not enhanced in response to exogenous auxin treatment.

## 3. Discussion

### 3.1. Response of Fra a to Auxin

Strawberry is a non-climacteric fruit and ripening is regulated by various plant hormones [24]. Among these phytohormones, auxin has long been considered to be an important hormone controlling both fruit fleshiness and ripening based on the landmark study by Nitsch [11]. The endogenous auxin indole-3-acetic acid (IAA) is biosynthesized in achenes and subsequently influences growth of the receptacle. Given that IAA is easily degraded by light [25], the synthetic auxin NAA was used for the present long-term experiment in the greenhouse. The same phenomena reported by Nitsch were observed in strawberry fruit following achene removal and NAA application to fruit as a lanoline paste (−Achene+NAA in Figure 1). In the absence of auxin, normal fruit were not developed (Figure S1). This result was consistent with previous findings [11,26], and supports the conclusion that auxin is required for strawberry fruit growth and ripening. We hypothesized that Fra a are responsive to auxin for their function as PR-10. However, NAA application did not alter the expression of Fra a at both transcriptional and translational levels. Surprisingly, enhanced induction of Fra a proteins was observed only in the “−Achene+NAA” treatment. We analyzed *FaAux/IAA* transcript levels by real-time PCR to examine the efficacy of exogenous auxin application on strawberry fruit development. The *Aux/IAA* gene family encodes short-lived nuclear proteins that function as transcriptional regulators to modulate plant responses to auxin [27,28]. Liu et al. [29] reported that accumulation of *FaAux/IAA* transcripts increases in strawberry fruit following application of NAA. *FaAux/IAA* expression increased in response to NAA treatment, especially at the red stage (Figure 3C), which was indicative of activation of the auxin-response mechanism in the fruit. In contrast, the application of NAA in the absence of achenes did not enhance *FaAux/IAA* expression (Figure 3C). This finding indicated that the induction of Fra a by “−Achene+NAA” treatment was not caused by a direct effect of auxin, but by an indirect response to other phenomena.

The induction of Fra a by auxin was also tested by applying AOPP and PCIB as auxin inhibitors. AOPP and PCIB treatments also supported the conclusion that Fra a expression is not regulated by auxin. AOPP is an auxin biosynthesis inhibitor in *Arabidopsis* [30]. PCIB impairs the auxin-signaling pathway in *Arabidopsis* roots [31]. AOPP and PCIB did not significantly regulate *FaAux/IAA* expression (Figure 4C), thus, these inhibitors did not completely suppress the auxin metabolism pathway, but did inhibit fruit ripening (Figure 3 and Table 2). AOPP inhibits activity of l-phenylalanine ammonia-lyase (PAL), the enzyme in the phenylpropanoid biosynthesis pathway that regulates strawberry pigmentation [32,33]. Thus, the characteristics of fruit treated with AOPP might reflect the inhibition of PAL activity rather than the auxin biosynthesis pathway. In post-harvest treatments to analyze the short-term response, Fra a proteins were not responsive to auxin (Figure 5A,B). We concluded that exogenous NAA does not directly regulate Fra a expression in strawberry fruit. This might indicate that endogenous IAA is independent to Fra a induction. NAA could be useful for induction of parthenocarpic strawberry fruit without increasing Fra a protein expression in the fruit.

Other factors that may regulate Fra a include wounding response or fruit maturity. The surgical removal of achenes from the receptacle would be accompanied by wound stress. We were unable to evaluate the impact of achene removal in the present study because fruit development ceased in the absence of achenes. *PR-10* gene expression and protein accumulation in response to wounding has been reported in several plant species [19,34]. In such cases, the transient mechanical damage should be considered. It is also important to note that allergens may be readily induced by environmental factors.

### 3.2. Fra a Expression Profile during the Ripening Process

*Fra a* transcript levels differ at each fruit ripening stage in strawberry cv. “Camarosa” [4]. In the present analysis of cv. “Akihime”, similar expression patterns were observed, with *Fra a 1* downregulated and *Fra a 2* upregulated during the progression of fruit ripening (Figure 2A,B). In response to pre-harvest auxin treatments, the a* score and *Fra a 1* transcript level showed a weak negative correlation (*R*^2^ = 0.31), whereas those of *Fra a 2* were positively correlated (*R*^2^ = 0.69). The auxin inhibitor treatments delayed fruit ripening (Figure 3) and 100 µM PCIB significantly induced *Fra a 1* gene expression (Figure 4A). The fruit ripening process, per se, seems a trigger of the *Fra a* gene expression.

The treatment “–Achene+NAA” promoted fruit ripening from the large green stage (Figure 1 and Table 1), however, only Fra a 2 translational levels were changed during ripening (Figure 2D,E). The enhanced induction of Fra a 2 protein by “–Achene+NAA” treatment may be caused by fruit maturation progresses. IAA levels in the fruit rise rapidly at an early developmental stage and then decline as ripening progresses [23]. In contrast, abscisic acid levels are lowest in young fruit and increase as the fruit ripens [35,36]. Gibberellin and brassinosteroids are also associated with fruit ripening [37,38]. Thus, phytohormones other than IAA may be associated with fruit maturation and induction of Fra a 2 gene/protein. Elucidation of the regulatory mechanism of Fra a expression may enable its control by exogenous treatment with an inhibitor.

In the present study, Fra a 1 protein accumulation pattern did not correspond with changes in either the *Fra a 1* transcript level or the a* score in all treatments. The reason for this may be temporal differences between mRNA and protein degradation. In mammalian cells, the average half-life of RNA is shorter than that of protein [39]. In *Arabidopsis*, the rate of protein degradation varies at different developmental stages [40].

In conclusion, Fra a gene and protein expression in strawberry fruit is not regulated directly by auxin, but more likely by ripening metabolic processes or wounding stress in a paralog-specific manner. To control Fra a accumulation in strawberry fruit, it will be important to regulate Fra a expression at the appropriate developmental stage to be consistent with the defense mechanisms of the PR-protein. In addition, Fra a mRNA and protein expression patterns scarcely correspond at the ripening stage. In order to suppress translational products, silencing prior to gene expression is required, but the appropriate stage will differ between paralogs: at the small green stage for *Fra a 1* and at the red stage for *Fra a 2*. In addition, monitoring of Fra a protein accumulation to evaluate allergenic capacity is required.

## 4. Materials and Methods

### 4.1. Plant Materials

Strawberry plants (*Fragaria* × *ananassa* cv. “Akihime”) were grown in containers in a glasshouse at Kobe University, Japan. Fruiting was promoted by artificial pollination of flowers. Fruit ripening stages were defined as small green, large green, white, and red. The fruit appearance, detailed definition, and approximate number of weeks after pollination at each stage were defined as shown in Figure 1. After sampling and characterization, fruits were immediately frozen in liquid nitrogen, and ground to a fine powder using a Multi-beads Shocker (Yasui Kikai, Osaka, Japan) at 2000 rpm twice for 10 s. Each powdered sample was stored at −80 °C until extractions of RNA and protein.

### 4.2. Pre-Harvest Treatment

For long-term treatment of fruit, the synthetic auxin NAA was used owing to its persistence and resistance to degradation relative to IAA. Crystals of NAA were dissolved in lanolin solution as described by Serrani et al. [41]. The final concentration of NAA in the lanolin solution was adjusted to 3000 µM. AOPP and PCIB were prepared in the same manner at concentrations of 100, 1000, or 2000 µM. The control consisted of lanolin solution without NAA or inhibitors. At the small green or white stage, depending on the experimental design, the entire surface of the fruit was pasted with the respective solution. For achene removal treatment, achenes were excised from the receptacle at the small green stage with a knife before pasting with NAA solution. Treated fruit were sampled at each developmental stage and characterized.

### 4.3. Post-Harvest Treatment

Crystals of IAA were dissolved in a small amount of ethanol with ultrasonic treatment, and diluted to 3000 µM in ultrapure water. Ethanol solution (0.3%, *v*/*v*) was used as a control treatment. Fruit at the large green, white, and red developmental stages were packed into a plastic box and immersed in the respective solution. Dipped fruit were covered with aluminum foil and incubated for 1, 2, 5, 10, or 24 h at 20 °C. After incubation, the fruit were frozen as described in Section 4.1. Non-treated fruit were used as the 0-h sample.

### 4.4. Characterization of Fruit

A Handy Spectrophotometer NF 333 (NIPPON DENSHOKU, Tokyo, Japan) was used for quantification of fruit color. The L*, a*, and b* scores were averaged from three random measurements. L* represented the lightness (black [0] and white [100]), a* represented the color difference between red (positive values) and green (negative values), and b* represented the color difference between yellow (positive values) and blue (negative values). A digital DT-100 vernier caliper (Niigata seiki, Niigata, Japan) was used to measure both longitudinal and transverse diameters of the fruit. The fresh weight of the fruit (receptacle and achenes) without the calyx was determined with a digital precision balance FX-1200i (A and D, Tokyo, Japan).

### 4.5. RNA Extraction and Quantitative Real-Time PCR

RNA was extracted using a Maxwell^®^ 16 Automated Purification System (Promega, Madison, WI, USA) with Fruit-mate™ for RNA Purification (Takara Bio, Kusatsu, Japan). The RNA concentration was determined with a Nanodrop 1000 spectrophotometer (Thermo Fisher Scientific, Waltham, MA, USA), and stored at −80 °C. 

For reverse transcription we used 100 ng total RNA with the ReverTra Ace^®^ qPCR RT Kit (Toyobo, Osaka, Japan) in accordance with the manufacturer’s protocol. The cDNA solution was diluted two-fold and stored at −20 °C. 

Real-time PCR was carried out in a LightCycler Nano or 480 (Roche Diagnostics, Basel, Switzerland) with the THUNDERBIRD^®^ SYBR^®^ qPCR Mix (Toyobo). The PCR assay was prepared to 20 µL total volume containing 10 µL THUNDERBIRD^®^ SYBR^®^ qPCR Mix, 2.0 µL cDNA solution, and 10 µM gene-specific primers. The gene-specific primer sequences are listed in Table S1. The thermal cycling procedure was as follows: 95 °C for 30 s, three-step amplification consisting of 40 cycles of 95 °C for 15 s, 60 °C for 30 s, and 72 °C for 30 s, a pre-melt hold at 95°C for 10 s, and melting at 60 °C to 97 °C at a rate of 0.1 °C/s. All target gene data were normalized against *EF1α* transcript levels.

### 4.6. Protein Extraction and SDS-PAGE

Protein was extracted using a modified phenol extraction protocol [42]. From 1.0 g powdered fruit, soluble protein was extracted with 5 mL extraction buffer mix (0.5 M Tris–HCl adjusted to pH 8.8, 0.1 M KCl, 0.7 M sucrose, 0.1× protease inhibitor cocktail (Nacalai Tesque, Kyoto, Japan), 1.0 mM phenylmethylsulfonyl fluoride, 50 mM ethylenediaminetetraacetic acid, 1.0% (*w*/*v*) polyvinylpyrrolidone, 2.0% (*v*/*v*) β-mercaptoethanol). The mixture was added to 5 mL Tris-buffered phenol (pH 8.0), sufficiently vortexed and centrifuged at 3901× *g* for 10 min at 4 °C. The supernatants were transferred to a new tube, and extracted again using an equal volume of extraction buffer mix. Protein was extracted from the supernatant using 0.1 M ammonium acetate in cold MeOH overnight at −20 °C. The centrifuged pellet was washed three times with the same solution. The final pellet was vacuum dried and dissolved in buffer for SDS-PAGE (50 mM Tris–HCl adjusted to pH 6.8, 0.25% (*w*/*v*) SDS, and 10% (*v*/*v*) glycerol). The protein concentration was quantified with a Qubit 2.0 fluorometer (Thermo Fisher Scientific) and stored at −20 °C.

Sample solution including 1–2 µg protein was separated in a 15% acrylamide gel. After electrophoresis, the acrylamide gel was stained with Coomassie Brilliant Blue Stain One Super (Nacalai Tesque) in accordance with the kit instructions (Figure S2).

### 4.7. Immunoblotting

Separated proteins were blotted on polyvinylidene difluoride membranes by semi-dry transfer. Target proteins were detected with the antibodies as follows: anti-Fra a 1e with 6× His (1:400 dilution) and alkaline phosphatase-conjugated anti-guinea pig IgG (1:3000 dilution; ab102367, Abcam, Cambridge, UK) were used to identify Fra a 1. The anti-Fra a 2 synthetic peptide (1:500 dilution) and alkaline phosphatase-conjugated anti-rabbit IgG (1:2000 dilution; S373B, Promega) were used to specifically identify Fra a 2. The BCIP-NBT Solution Kit (Nacalai Tesque) was used for alkaline phosphatase staining.

### 4.8. Statistical Analyses

Statistical analyses were performed using JMP 12 and 13 (SAS Institute, Cary, NC, USA). Significant differences were determined by Tukey–Kramer’s HSD test or the Wilcoxon signed rank test depending on the normality of the data. The data were the mean ± standard error of four biological replicates.

## Figures and Tables

**Table 1:** Development and maturation of strawberry (*Fragaria* × *ananassa* cv. “Akihime”) fruits in response to pre-harvest auxin treatment.

Treatment ^z^	L* ^y^		a* ^y^			b* ^y^		LD ^x^ (mm)		TD ^x^ (mm)			FW ^x^ (g)	
*Large green*														
Control	57.7 ± 2.8	a ^w^	−11.8 ± 4.0		b	26.6 ± 1.6	a	22.2 ± 0.7	a	14.8 ± 0.9	a		2.1 ± 0.4	a
−Achene+NAA	59.6 ± 2.3	a	4.8 ± 2.5	a		28.3 ± 2.0	a	22.4 ± 0.6	a	13.1 ± 1.3	a		1.9 ± 0.3	a
+NAA	56.9 ± 1.8	a	−7.5 ± 3.1	a	b	28.3 ± 1.1	a	24.3 ± 2.0	a	15.5 ± 2.0	a		2.7 ± 0.8	a
*White*														
Control	62.0 ± 1.5	a	−0.6 ± 3.3	a		24.6 ± 2.0	a	27.9 ± 2.4	a	17.8 ± 1.1	a	b	3.9 ± 0.9	a
−Achene+NAA	62.2 ± 1.9	a	4.0 ± 9.1	a		24.3 ± 0.7	a	22.8 ± 1.7	a	15.2 ± 1.0		b	2.8 ± 0.3	a
+NAA	59.4 ± 1.6	a	−2.1 ± 3.0	a		23.4 ± 0.2	a	29.2 ± 1.8	a	19.7 ± 1.0	a		4.5 ± 0.7	a
*Red*														
Control	40.6 ± 3.5	a	35.1 ± 2.8	a	b	26.1 ± 1.6	a	35.1 ± 1.8	a	22.8 ± 1.6	a		8.3 ± 1.0	a
−Achene+NAA	40.6 ± 3.2	a	38.4 ± 2.4	a		24.4 ± 3.4	a	29.8 ± 1.9	a	21.7 ± 0.9	a		6.9 ± 1.1	a
+NAA	45.2 ± 3.2	a	33.1 ± 3.7	a	b	25.7 ± 0.2	a	38.6 ± 3.2	a	25.9 ± 2.0	a		10.8 ± 2.7	a
+NAA in white	48.4 ± 5.6	a	23.7 ± 5.8		b	20.4 ± 1.2	a	37.4 ± 3.9	a	26.5 ± 1.0	a		10.3 ± 1.4	a

^z^ Auxin treatments were applied at the small green or white stage as shown in Figure 1. Fruit were harvested at the large green, white, and red stages, respectively. ^y^ L*(lightness), a* (redness-greenness), and b* (yellowness-blueness) scores were measured in triplicate with a Handy Spectrophotometer NF 333 (NIPPON DENSHOKU, Tokyo, Japan). Data are the mean ± standard error of four independent fruit. ^x^ Both longitudinal (LD) and transverse diameters (TD), and fresh weight (FW) were measured with a digital vernier caliper DT-100 (Niigata seiki, Niigata, Japan), and a digital precision balance FX-1200i (A and D Company, Limited, Tokyo, Japan), respectively. ^w^ Different letters among treatments following values indicate a significant difference (Tukey–Kramer’s HSD test, *n* = 4, *p* < 0.05).

**Table 2:** Development and maturation of strawberry (*Fragaria* × *ananassa* cv. “Akihime”) fruit in response to pre-harvest treatment with the auxin inhibitors AOPP and PCIB.

Treatment ^z^	L* ^y^			a* ^y^			b* ^y^		LD ^y^ (mm)		TD ^y^ (mm)		FW ^y^ (g)	
*AOPP*														
Control	42.8 ± 1.7		b ^x^	30.4 ± 1.7	a		21.6 ± 2.1	a	34.0 ± 2.7	a	22.6 ± 0.8	a	6.8 ± 0.6	a
100 μM	45.0 ± 1.9		b	24.1 ± 2.3	a		20.7 ± 0.7	a	33.3 ± 3.5	a	23.3 ± 1.5	a	7.4 ± 1.4	a
1000 μM	59.1 ± 3.0	a		11.3 ± 2.5		b	25.5 ± 2.0	a	29.8 ± 3.8	a	19.8 ± 2.5	a	5.8 ± 1.8	a
*PCIB*														
Control	40.2 ± 1.1		b	31.2 ± 0.8	a		24.5 ± 1.7	a	32.5 ± 1.9	a	24.2 ± 1.4	a	7.3 ± 1.2	a
100 μM	57.4 ± 3.8	a	b	0.6 ± 3.8		b	20.0 ± 2.0	a	26.5 ± 2.1	a	21.1 ± 2.0	a	5.2 ± 0.6	a
1000 μM	53.4 ± 4.5	a	b	11.8 ± 9.7		b	21.9 ± 1.8	a	25.7 ± 2.4	a	19.4 ± 2.2	a	4.8 ± 0.9	a

^z^ Auxin treatments were applied at the small green stage, as shown in Figure 3. Fruit were harvested at approximately 5 WAP. ^y^ All data were determined as described in Table 1. ^x^ Different letters among treatments following values indicate a significant difference (Tukey–Kramer’s HSD test, *n* = 4, *p* < 0.05).

## Supplementary Materials

Supplementary materials can be found at www.mdpi.com/1422-0067/18/6/1186/s1.

## Abbreviations

AOPP	l-2-Aminooxy-3-phenylpropionic acid
EF1α	Elongation factor 1α
IAA	Indole-3-acetic acid
NAA	1-Naphthaleneacetic acid
OAS	Oral allergic syndrome
PCIB	*p*-Chlorophenoxyisobutyric acid
PR-10	Pathogenesis-related 10
ROD	Relative optical density
WAP	Weeks after pollination
