# Supplementary material for: Expression Profiling of Strawberry Allergen Fra a during Fruit Ripening Controlled by Exogenous Auxin
Source: Int J Mol Sci. 2017 Jun 2;18(6):1186. doi: 10.3390/ijms18061186 (PMC5486009; doi:10.3390/ijms18061186)
Supplement: Supplementary file 1 [file ijms-18-01186-s001.pdf]

Expression Profiling of Strawberry Allergen Fra a during Fruit Ripening Controlled by Exogenous Auxin

Misaki Ishibashi, Hiroki Yoshikawa and Yuichi Uno

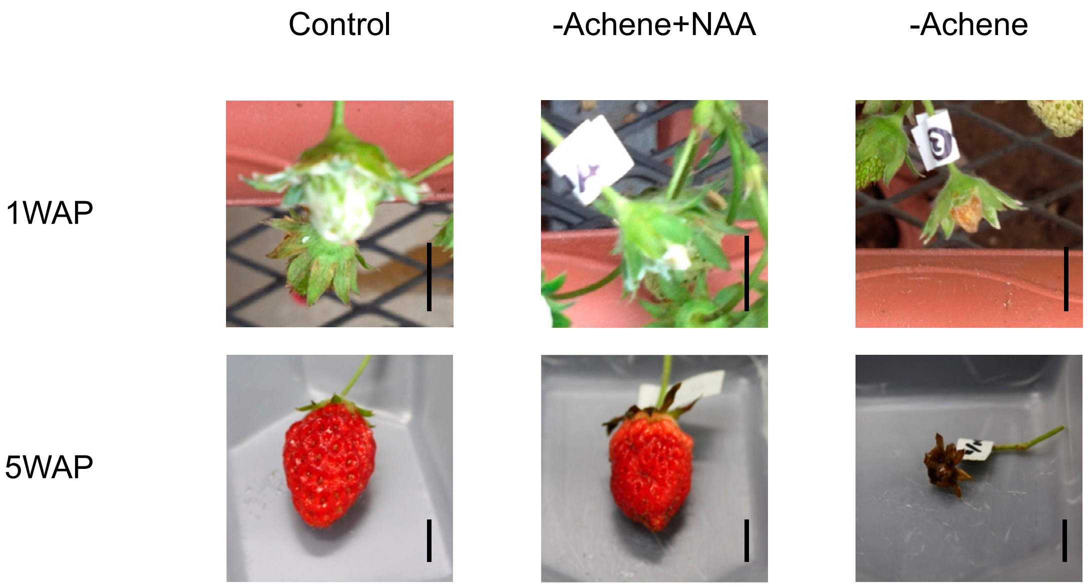

Figure S1. Development of strawberry (*Fragaria* × *ananassa* cv. 'Akihime') fruit in response to pre-harvest treatment with auxin. Strawberry plants were grown in containers in a glasshouse. Approximate number of weeks after artificial pollination (WAP) is shown on the left of the photographs. The surface of the fruit was pasted with lanolin emulsion at the small green stage. The lanoline emulsion contained solvent alone (Control). Achenes were removed and pasted 3000 μM 1-naphthaleneacetic acid (−Achene+NAA) or lanolin solvent alone (−Achene). Scale bar represents 1.0 cm.

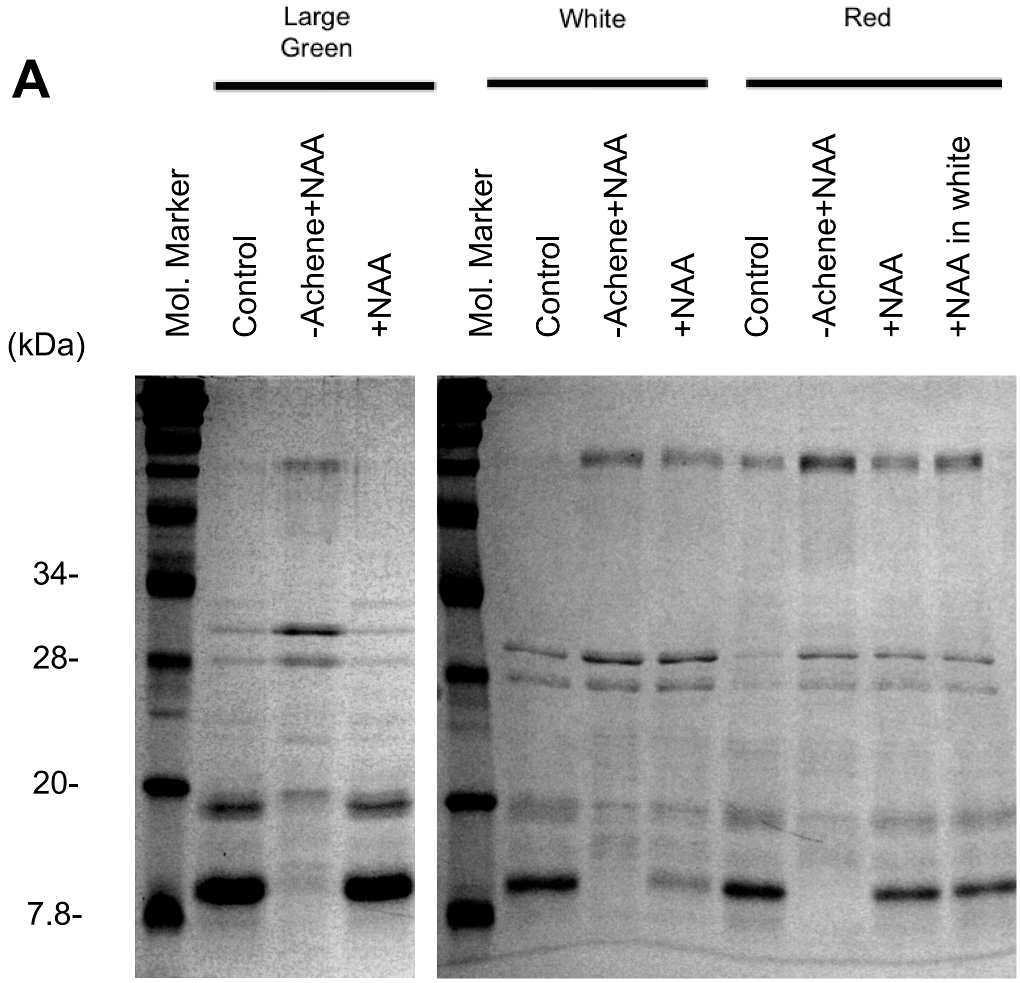

Figure S2A. Each profile of protein in strawberry fruit (*Fragaria* × *ananassa* cv. 'Akihime'). Culture and pre-harvest treatments were performed as described in Figure 1. Protein extraction was performed as described in Figure 2. 2μg protein was separated by SDS-PAGE with 15% acrylamide gels, and stained with Coomassie brilliant blue solution (Nacalai Tesque).

B

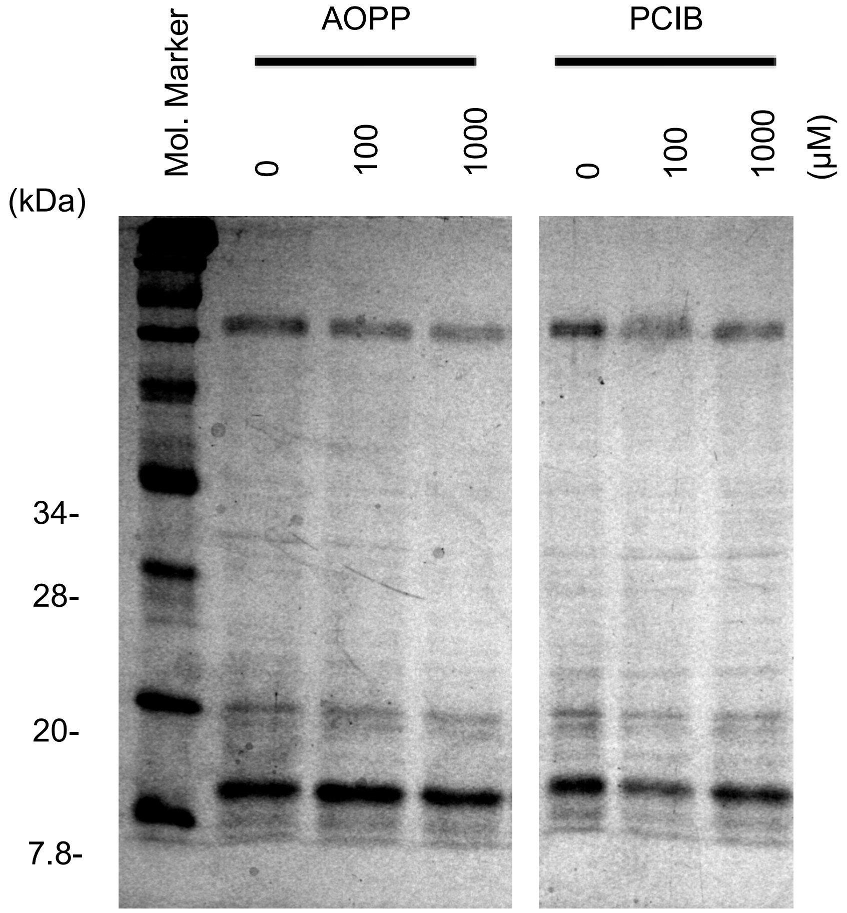

Figure S2B. Each profile of protein in strawberry fruit (*Fragaria* × *ananassa* cv. 'Akihime'). Culture and pre-harvest treatments were performed as described in Figure 3. Protein extraction was performed as described in Figure 4. 2μg protein was separated by SDS-PAGE with 15% acrylamide gels, and stained with Coomassie brilliant blue solution (Nacalai Tesque).

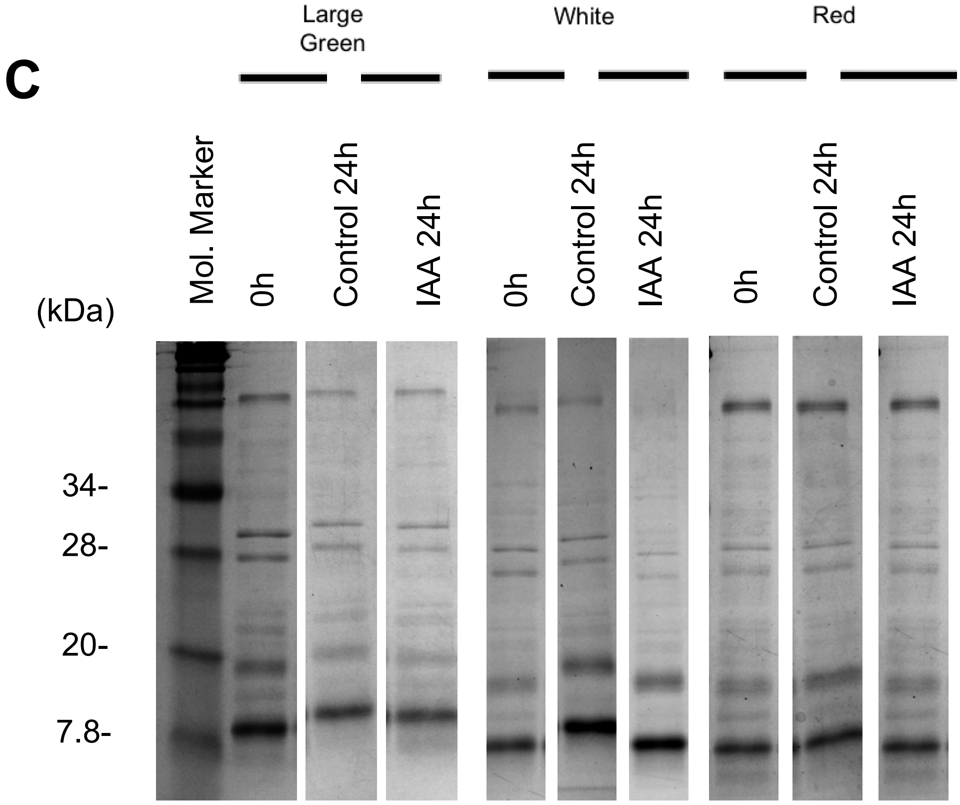

Figure S2C. Each profile of protein in strawberry fruit (*Fragaria* × *ananassa* cv. 'Akihime'). Culture and pre-harvest treatments were performed as described in Figure 5. Protein extraction was performed as described in Figure 5. 2μg protein was separated by SDS-PAGE with 15% acrylamide gels, and stained with Coomassie brilliant blue solution (Nacalai Tesque).

Table S1. Primer sequences in real-time PCR.

Name	Sequence	Reference
Fra a 1-Forward	CACACCAAGGGAGATGTCG	
Fra a 1-Reverse	GGGTGGTCCTTGAGGTATCC	
Fra a 2-Forward	ACACCAAAGGTGACGTGGA	
Fra a 2-Reverse	ATTAGGATTGGCCAAGAGGTAG	
FaAux/IAA-Forward	AAAGCGGTGGGATGTTCGTG	
FaAux/IAA-Reverse	CCTTGAGCAGCTCTGGATATCC	
EF1α-Forward	TGGATTTGAGGGTGACAACATGA	[1]
EF1α-Reverse	GTATACATCCTGAAGTGGTAGACGGAGG	[1]

1. Amil-Ruiz, F.; Garrido-Gala, J.; Blanco-Portales, R.; Folta, K.M.; Muñoz-Blanco, J.; Caballero, J.L. Identification and Validation of Reference Genes for Transcript Normalization in Strawberry (*Fragaria* × *ananassa*) Defense Responses. *PLoS ONE* **2013**, *8*, e70603.
